# A Latent Profile Analysis of COVID-19 and Influenza Vaccine Hesitancy among Economically Marginalized Hispanic Mothers of Children under Five Years of Age in the US

**DOI:** 10.1007/s40615-024-02012-1

**Published:** 2024-05-07

**Authors:** Yea Won Park, Elise Bragard, Purnima Madhivanan, Celia B. Fisher

**Affiliations:** 1 Department of Psychology. Dealy Hall, Fordham University, 441 E Fordham Rd, Bronx, NY 10458 USA; 2 Health Department of Public Health Sciences, University of Connecticut, 195 Farmington Avenue, Farmington, CT 06032 USA; 3 Department of Health Promotion Sciences, University of Arizona, Mel and Enid Zuckerman College of Public Health, 1295 N. Martin Ave. Tucson, Tucson, AZ 85721 USA

**Keywords:** Hispanic, COVID-19, Influenza, Vaccination, Pediatric, Cultural Health Beliefs Model, acculturation, medical mistrust

## Abstract

Rates of COVID-19 and influenza vaccine coverage among Hispanic young children continue to be low in comparison to other racial and ethnic groups in the United States. This study utilized a person-centered approach to understand COVID-19 and influenza vaccination hesitancy for young children under the age of five among 309 economically marginalized Hispanic mothers. Drawing on the cultural health belief model, in 2022, following FDA approval of the COVID-19 vaccine for young children, a latent profile analysis was conducted from which three profiles emerged. The *Low Acculturation* group (Profile 1), was notable for lower acculturation, moderate cultural medical mistrust, lower access to vaccines, and higher financial security. Compared to Profile 1, the two remaining profiles had higher acculturation and lower levels of financial security, but differed in that the *High Acculturation* group (Profile 2) had higher vaccine accessibility and the *Moderate Acculturation* group (Profile 3) had higher cultural medical mistrust. Relative to other profiles, *Low Acculturation* mothers were more likely to plan to vaccinate their child against current and seasonal COVID-19 and seasonal influenza, report that their child’s health provider recommended the COVID-19 shot and reported lower COVID-19 and influenza vaccine mistrust. However, they also reported lower vaccine accessibility and moderate levels of cultural medical mistrust. The study highlights the importance of developing person-centered public health strategies that draw on Hispanic cultural values and consider diversity within lower income Hispanic populations to increase future pediatric COVID-19 and flu vaccination coverage among young Hispanic children.

## Introduction

Since June 2022 when the U.S, Food and Drug Administration (FDA) approved COVID-19 vaccination for children under 5 years of age, public health campaigns have been initiated to increase pediatric COVID-19 vaccination among hard-to-reach populations. These efforts build upon prior strategies aimed at increasing seasonal influenza vaccinations among young children, especially those living in economically marginalized communities [1–4]. The Centers for Disease Control and Prevention (CDC) recommends that for COVID-19, unvaccinated children aged 6 months to 4 years receive either a 2-dose series of updated Moderna (2023–2024 Formula) or a 3-dose series of updated Pfizer-BioNTech (2023–2024 Formula), while previously vaccinated children should follow guidelines for updated doses with recommended intervals (e.g., children previously vaccinated with 1 dose of Moderna should receive 1 updated dose of Moderna 4–8 weeks after the most recent dose [5]). Additionally, annual influenza vaccination is recommended for individuals aged 6 months and older, with a second dose advised for children aged 6 months through 8 years who have not previously received two doses or are uncertain about their past vaccinations, to be given at least 4 weeks after the initial dose regardless of previous season dosing [6].

However, despite public health efforts, pediatric COVID-19 and influenza vaccination remain low in underserved Hispanic communities. For example, a report on flu vaccination coverage during the 2022–2023 influenza season indicated a suboptimal 67.4% vaccination rate among Hispanic children between the ages of 6 months and 4 years [7]. According to the CDC, COVID-19 vaccination rates for Hispanic children during 2023–2024 were slightly higher than those for Black children but lower than rates among Non-Hispanic White and Non-Hispanic other children[8]. Moreover, a U.S. national survey found that an educational level lower than a bachelor’s degree and income less than 400% below the U.S. poverty level (e.g., $124,800 annual income for a family of four in 2024) predicted influenza vaccine hesitancy [9]. According to CDC data from February 17, 2022, among children under five years of age, only 19.9% of Hispanic children received at least one COVID-19 vaccination dose (despite the Hispanic population accounting for 25.9% of the US population [10]). Overall, larger proportion of parents say they are confident in the safety of the flu vaccine (68%) compared to the COVID vaccine (48%) [11]. Moreover, vaccination rates for both COVID-19 and influenza, to date, are particularly low in border states such as Texas (COVID-19: 11.4%; Influenza: 38.6%), Arizona (15.8%; 42.1%), and New Mexico (22.3%; 46.9%), where those with Hispanic heritage make up a considerable portion of the population [7, 12].

Given the continuing low COVID-19 vaccination rates among young Hispanic children and the declining rates of influenza vaccination during the COVID-19 pandemic, there is a need to better understand socio-cultural determinants influencing parental vaccine hesitancy among lower-income economically marginalized Hispanic parents. Culture has a paramount role in understanding health behavior and health disparities [13]. The cultural health belief model [14] highlights the importance of considering the influence of cultural beliefs and structural factors (e.g., economic marginalization) on health outcomes. For instance, cultural values (*familism, respecto, confianza*) have been reported to have a buffering effect on various health behaviors among Hispanic adult samples [15–18]. However, the protective effect of Hispanic cultural values on health behavior is noted to decrease with greater years of living in the US [19, 20], in which acculturation to Anglo activities and values may play a role.

In studies that apply the cultural health belief model [14], acculturation and cultural medical mistrust are factors influencing vaccination resistance among parents. Acculturation is a multidimensional process that refers to the cultural adaptation of Hispanic individuals living in the US to adhere to and adopt the mainstream host culture [21]. Individuals of Hispanic heritage differ in the extent to which they retain their values and cultural practices from their home countries and endorse the values and practices of their host culture. Years of living in the U.S. is often used as a proxy for acculturation and commonly used measures for acculturation include language use and preferences, as well as one’s psychological attachment to and belonging within the Anglo-American and Hispanic cultures [22, 23].

One study reported that among Mexican American mothers in Texas, those who were more acculturated had lower favorable attitudes toward pediatric immunization (i.e., combination of oral polio, measles-mumps-rubella, diphtheria-tetanus-pertussis vaccines) and reported lower parental responsibility for up-to-date child’s immunization status [24]. Acculturation may have a similar influence on vaccine hesitancy towards pediatric influenza and COVID-19 among Hispanic caregivers. In addition, cultural medical mistrust, the belief that those of Hispanic descent will receive poorer medical care may stem from language differences, past discrimination experiences, and influence future anticipation of discrimination of Hispanic patients from healthcare providers [25]. Several studies have reported cultural medical mistrust as a risk factor for vaccine acceptance against COVID-19 among Hispanic families [26, 27], and seasonal influenza vaccination uptake among low-income adults that included samples of Hispanic respondents [28].

Structural factors, such as accessibility challenges to vaccination and financial security also play a role in Hispanic health behaviors [29–31]. Accessibility challenges to vaccination refer to practical obstacles such as job or family responsibilities, lack of transportation, difficulty finding affordable healthcare providers, and a lack of vaccination infrastructure. Economically marginalized Hispanic families may find it difficult to take leave from work to get their children vaccinated or have limited access to primary care providers or vaccination locations [10]. A meta-analysis that examined barriers to influenza vaccination intention in the US and European countries, found inconvenience due to lack of transportation and the expense of vaccination were significant barriers to influenza vaccination uptake [30]. However, one study reported that perceived barriers (e.g., time/money) were unrelated to vaccination intentions for COVID-19 among an Israeli sample [29]. Various studies have reported links between economic burden (financial strain, insurance coverage) and vaccination uptake against influenza and COVID-19 [25, 32–34]. To date, there is a paucity of research on whether accessibility relates to pediatric vaccination uptake against COVID-19 and influenza among economically marginalized Hispanic populations in the U.S.

Despite theoretical perspectives [13, 14] that emphasize that health behavior is influenced by multi-dimensional factors, with few exceptions, most research on vaccine uptake has relied on a variable approach that looks at factors associated with vaccine hesitancy across members of a population rather than a person-centered approach that seeks to understand differences within a population (e.g., 27, 28). For instance, acculturation, cultural medical mistrust, accessibility, and financial security may be correlated with vaccine acceptance across Hispanic parents when assessed as a group. However, a person-centered approach may reveal a relationship between vaccine hesitancy and distinct differences in how these variables are proportioned among different Hispanic parents. Specifically, one group of economically marginalized Hispanic mothers may be highly acculturated, but also have high levels of cultural medical mistrust, financial insecurity, and low vaccine accessibility. On the other hand, another group may have lower acculturation and lower cultural medical mistrust, but have high financial insecurity and vaccine accessibility [35].

The few studies that have utilized a person-centered approach to assess associations between profiles and influenza and COVID-19 vaccination uptake, have been restricted to adult or college students. For example, a study involving a sample of predominantly female Hispanic and Black (25.1%) adults in South Florida found higher COVID-19 vaccination rates in respondents reporting three distinct profiles based on trust in various health information sources including doctors and community leaders [36]. Additionally, among a diverse global sample, Howard and colleagues [37] found evidence of eight vaccine hesitancy profiles and reported that individuals who were more suspicious of health risks associated with the vaccine (i.e., Distrusting profile) and those who had greater accessibility challenges (i.e., Contrarian profile) were less likely to report current and future vaccination intent for influenza and COVID-19. Though these studies suggest that the person-centered methodology advances the vaccine hesitancy literature, there are limitations due to utilizing combined U.S. ethnic and international samples and only examining adult vaccination uptake. Moreover, because parents report being more hesitant to vaccinate young children [34], work is needed to determine whether socio-cultural profiles of Hispanic mothers relate to pediatric vaccination intent for COVID-19 and influenza.

The present study applied a person-centered approach, to identify potentially unique profiles among economically marginalized Hispanic parents relevant to their willingness to vaccinate their young children against COVID-19 and influenza infection. We accomplished this through a latent profile analysis (LPA; [38]) on variables associated with acculturation, cultural medical mistrust, accessibility, and financial security. Then we investigated relationships between profiles and attitudes related to current and seasonal pediatric COVID-19 and influenza vaccinations and other correlates including age of child [39, 40], insurance [41], income [39], COVID-19 vaccine mistrust [42], and flu Vaccine mistrust [43]. Based on prior research, we expected that relative to other profiles, the profile with lower acculturation and cultural medical mistrust and higher levels of accessibility and financial security would report greater intent for both COVID-19 and influenza vaccination uptake.

### Method

#### Procedure and Participants

Recruitment and data collection were conducted through Qualtrics XM, a survey aggregator that recruits individuals who sign up to take paid surveys. Participants had the option to complete the online Qualtrics survey in English or Spanish (1.6% chose the Spanish version). Individuals who clicked on a link describing a survey related to children’s health viewed a screener, and those who qualified were able to access an informed consent page which described the study in detail. Of the 1216 who responded to the screener, 490 met inclusion criteria, 7 did not provide consent, 112 did not complete the survey, and 62 failed attention and validity checks. The final sample size of 309 Hispanic female guardians (≥ 21 years old) met the suggested minimum sample size for adequate power with LPA analysis [44]. Data were collected over the course of two weeks in 2022 (July 14 through July 31) as part of a larger study [45], following the June 17, 2022 FDA emergency approval of the COVID-19 vaccine for children six months to four years of age. Inclusion criteria included living in the US for more than 5 years, being a Hispanic female guardian, having a child between ages 1–4, having a household income under $55,000, and residing in one of three border states: Arizona, Texas, and New Mexico. The final survey protocol was reviewed and revised by a Hispanic health advocacy group and approved by the University’s institutional review board IRB ID2176.

### Measures

#### Acculturation

Acculturation was measured with three indicators (behavioral acculturation, psychological acculturation, and length of stay in the US). The 6-item Short Acculturation Scale for Hispanics (SASH; [46]) measured behavioral acculturation through language preferences (1 = Spanish Only to 5 = English Only) across multiple settings, including home, with friends, TV/movies/ radio/ online programs/ news coverage, and usual and preferred language with doctor. Scale scores were computed as the mean of all items and higher scores indicated greater acculturation. The 4-item Psychological Acculturation Scale (PAS; [23]) measured psychological acculturation. Participants rated their sense of belonging with 1 = Hispanic/Latino culture, 2 = both Hispanic/Latino culture and Anglo-American culture, or 3 = Anglo-American culture. An example item was “In your opinion which group of people best understands your ideas, your way of thinking?” Mean scores were calculated with the four items and higher scores indicated greater acculturation towards the Anglo-American culture. For the current study the inter-item reliabilities were *a* = 0.90 for SASH and *a* = 0.76 for PAS. For time living in the US participants rated one item on a 6-point Likert-type scale (1 = 1–2 years, 2 = 3–4 years, 3 = 5–10 years, 4 = 11–15 years, 5 = 16–20 years, 6 = Over 20 years). As indicated above, only individuals living 5 or more years in the US were included in the sample.

#### Cultural Medical Mistrust

Cultural medical mistrust was assessed using the 5-item Group-Based Medical Mistrust Scale (GBMMS; [47]). Responding to our advisory board’s recommendation that Hispanic individuals in these states often seek services from their pharmacists, participants rated their trust levels of both doctors and pharmacists towards Hispanic patients on a 6-point Likert-type scale (1 = Strongly disagree to 6 = Strongly agree). An example item is “I have personally been treated poorly or unfairly by doctors because I am Hispanic/Latino.” Positive items were reversed scored, items from both scales were combined into a total average score, and greater scores indicate higher medical mistrust; *a* = 0.86.

#### Accessibility Challenges to Vaccination

Participants rated nine items on a 6-point Likert-type scale (1 = Strongly disagree to 6 = Strongly agree). Items were adapted from the Barriers to Treatment Participation Scale [48]. The adapted measure included items involving stressors/obstacles related to vaccination uptake, relationship with medical personnel, critical events, and perceived relevance of the vaccine. Example items include “It is difficult to find transportation to a doctor or pharmacy that offers vaccination for children.” “My other family responsibilities make it difficult for me to take my child for a vaccination.” Items were reverse scored such that greater scores indicated greater accessibility to vaccination. The reliability of the measure was, *a* = 0.85.

#### Perceived Financial Security

A single item assessed financial burden. Participants rated the question “How would you describe your current financial situation?” The item was scored on a 3-point Likert-type scale (1 = I cannot make ends meet; 2 = I have just enough; 3 = I am comfortable). Greater scores indicated higher perceived financial security.

#### Demographics

Demographic information included racial and ethnic background, household income, state of residence, the age and gender of the target child, and child’s health insurance coverage.

#### Provider’s recommendation for COVID-19 Vaccine

A single item assessed physician/pharmacist recommendations of the COVID-19 vaccine. Mothers responded with three options: the child’s doctor/pharmacist had recommended their child to receive the COVID vaccine, did not mention the vaccine, or recommended against vaccination. Items were recoded as 1 = Provider did not mention or recommended against the vaccine and 2 = Provider recommended vaccination.

#### COVID-19 Vaccination Mistrust

Five items adapted from studies of different age and ethnic groups [42, 49, 50] measured knowledge and misconceptions about the COVID-19 vaccine. Items were rated on a 6-point Likert-type scale (1 = Strongly degree to 6 = Strongly agree). An example item was “The COVID-19 shot might cause serious side effects or lasting health problems for my child”. Positive items were reverse scored. Higher scores indicated greater vaccination mistrust: *a* = .89.

#### Intent to Vaccinate Child Against COVID-19

A single item measured mothers’ current intention to vaccinate for COVID-19 after the following statement: “The FDA has approved the COVID-19 vaccine for children 6 months and older. The vaccine requires children to get at least 2 shots. Please check the statement that best fits your plans concerning giving your youngest child age 1 – 4 the COVID-19 vaccine now that it is approved by the FDA.” Response options ranged from 1 = I will definitely not have my child receive their first COVID-19 shot to 5 = I will definitely have my child receive their first COVID-19 shot. The scale was recoded into two categories (1 = Definitely not, probably not, or unsure;  2 = Probably or definitely will).

#### Intent to Annually Vaccinate Child Against COVID-19

A single item measured mother’s future intention to vaccinate their child if COVID-19 became a seasonal infection requiring an annual vaccine. “If COVID-19 were a seasonal disease (occurs every year), how comfortable would you feel including it with other routine vaccinations for your youngest child between 1–4 years recommended by your doctor?” Response options were rated on a 5-point Likert-type scale: 1 = I would definitely not give my child the COVID-19 shot every year to 5 = I would definitely give my child the COVID-19 shot every year. Responses were coded into 2 categories (1 = Definitely not, probably not, or unsure; 2 = Probably or definitely will).

#### Provider’s Recommendation for the Flu Vaccine

One item assessed doctor/pharmacist recommendations for the flu. Mothers responded with three options: the child’s doctor/pharmacist had recommended their child to receive the flu vaccine, did not mention the vaccine, or recommended against vaccination. Items were recoded as 1 = Provider did not mention or recommended against the vaccine and 2 = Provider recommended vaccination.

#### Flu Vaccination Mistrust

Five items adapted from studies involving different age and ethnic groups [42, 49, 50] measured knowledge and misconceptions about the flu vaccine. Items were rated on a 6-point Likert-type scale (1 = strongly degree to 6 = strongly agree). An example item included “The COVID-19/flu shot might cause serious side effects or lasting health problems for my child.” Positive items were reverse scored. Higher scores indicated greater vaccination mistrust, *a* = 0.81.

#### Intent to Vaccinate Child Against Influenza

Two items measured mothers’ intention to vaccinate their child against the flu. The item began with “The CDC recommends that children 6 months and older should receive a flu shot (flu vaccine) every year. Children 6 months- 8 years who are getting a flu shot for the first time should also get a second shot. Has your youngest child aged 1–4 years received their first flu shot?” Responses were rated on a 3-point Likert-type scale (1 = No, 2 = Unsure, and 3 = Yes). For mothers who selected either 1 = No or 2 = Unsure, an additional question was asked “Do you plan to have your child receive a flu shot?” The same ratings (1 = No, 2 = Unsure, and 3 = Yes) were utilized. Those who rated 3 = Yes for either the first or second question were recoded as current flu accepting (2 = Yes), whereas others (1 = No or 2 = Unsure) were recoded as current flu resistant (1 = No, Unsure).

#### Intent to Annually Vaccinate Child Against Influenza

A single item assessed mothers’ intention to vaccinate their child against seasonal influenza: “In the future do you plan to have your child receive the flu vaccine every year?” Responses were rated on a 3-point Likert-type scale that ranged from 1 = No, 2 = Unsure, and 3 = Yes. Items were recoded as 1 = No, Unsure and 2 = Yes.

### Analytical Plan

We conducted a 3-step Latent Profile Analysis with Mplus Version 8 [51] using the following indicator variables: (a) behavioral acculturation; (b) psychological acculturation; (c) years in US; (d) cultural medical mistrust; (e) vaccine accessibility; and (f) financial security. Four separate models were evaluated using a range of fit indices to determine the best fit of the data. Fit indices included the Akaike Information Criterion (AIC; [52]), Bayesian Information Criterion (BIC: [53]), adjusted BIC, entropy, Lo-Mendell-Rubin likelihood ratio test (LMR: [54]), ad hoc adjusted LMR ([54], and adequate class sizes (> 1%). Best fitting models are evaluated based on lower values of AIC and BIC, whereas the LMR and adjusted LMR indicated whether the model of interest had a significantly better fit than the model solution with one fewer class. Entropy indicates the confidence level of being classified into one group or another, which is recommended for the threshold level to be greater than 0.80 [55]. The R3step procedure within Mplus was utilized to investigate whether correlates influenced the likelihood of an individual belonging to one profile versus another. This approach uses multinomial logistic regression to examine associations between the correlates and extracted profiles. The maximum-likelihood with robust standard errors (MLR: [56]) was used. After identifying the profiles and correlates we assessed group differences through Multivariate Analysis of Variance and Chi Square analyses.

## Results

The mean age of Hispanic female guardians was 29.55 years old (SD = 6.71). Most participants (66.0%) resided in Texas, followed by Arizona (25.6%), and New Mexico (8.4%), most (68.6%) were born in the US and were of Mexican ethnicity (61.2%). The children’s age was equally distributed across one, two, three, and four years (*M*_child age_ = 32.72 months, *SD* = 13.81). Most children had either government or private insurance, and half the children received nutritional support from Supplemental Nutrition Assistance Program (SNAP). Less than half of the parents indicated their child had received all seven recommended routine vaccines [57]; however, between 9.4% and 21.7% indicated they did not know if their child had been vaccinated. Close to a third of respondents reported income under USD 20,000. Based on reported household size, 141 (45.6%) households met US poverty thresholds [58]. Approximately a quarter of the parents lived in rural areas, and a third lived in suburban and in urban areas.

**Figure 1:**
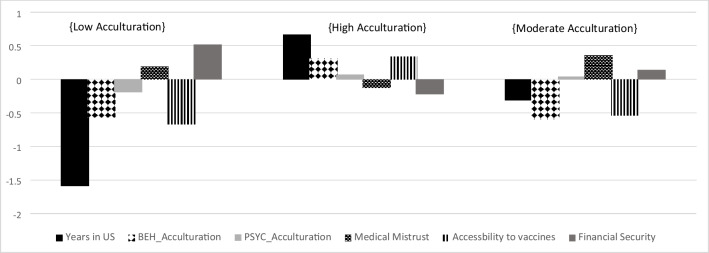
Standardized means for the three latent profiles: Profile 1 Low Acculturation (n = 79), Profile 2 High Acculturation (n = 202). Profile 3 Moderate Acculturation (n = 28)

Our analysis began by testing four theoretically viable profile models. All model fit indices are illustrated in Table 1. Based on the various model fit indices, we interpreted the 3-profile solution to be the best-fitting model for our data since it had the lowest AIC, BIC, and aBIC. In addition, the significant LMR and adjusted LMR tests demonstrated that the three-profile solution better fitted the model than the two-profile solution or the four-profile solution; the latter failed to converge, which indicated instability of profile extraction, and that four profiles were too many to extract. The three-profile solution included the most acceptable number of individuals in each profile, in which the smallest profile included 9.06%. The average probability for a participant’s most likely latent profile membership was 0.99 for the three-profile solution, which indicated high accuracy of class separation. Figure 1 provides a visual representation of the profile means. Relative to other profiles, Profile 1 was of moderate size (*n* = 79) and was notable for lower acculturation, moderate cultural medical mistrust, lower access to vaccines, and higher financial security. Profile 1 was labeled *Low Acculturation*. The two remaining profiles compared to Profile 1 had higher acculturation and lower levels of financial security, but differed in that Profile 2 had higher vaccine accessibility and Profile 3 had higher cultural medical mistrust. Profile 2, labeled *High Acculturation,* was the largest (*n* = 202) and was characterized by high acculturation, low cultural medical mistrust, high accessibility, and low financial security. Profile 3, labeled *Moderate Acculturation* was the smallest *(n* = 28) and was characterized by moderate acculturation, high cultural medical mistrust, low accessibility and moderate financial security.

**Table 1:** Model fit indices for competing latent profile models

Number of Profiles	AIC	BIC	aBIC	Entropy	LMR	*p*	Adjusted LMR	*p*	Smallest class size
1	5285.42	5330.22	5292.17						
2	4883.43	4954.27	4894.11	0.96	415.99	.00	405.88	.00	82(26.54%)
3	**4741.97**	**4839.04**	**4756.58**	**0.99**	**155.46**	**.02**	**151.68**	**.02**	**28(9.06%)**
4	Failed to converge								

*Note. n* = 309; The LMR test and the adjusted LMR test compare the current model to the model with k-1 profiles

Three separate multinomial logistic regression models were conducted to examine the relationships between the correlates and latent profiles. The first model included demographic characteristics (i.e., age of child, insurance, and income) as correlates. The second model included provider’s recommendation for the COVID-19 vaccine, COVID-19 vaccine mistrust, current and annual intentions of getting the COVID-19 vaccine for their child. The third model included provider’s recommendation for the flu shot, flu vaccination mistrust, and current and annual mothers’ intention of vaccinating their 1–5 year-old child against the flu.

Across the three profiles in each model, the following significant differences emerged. For the demographic correlates (model 1 in Table 2), significant relationships were found for child age and income. Moderate Acculturation mothers (Profile 3) had older children than High Acculturation mothers (Profile 2). Low Acculturation mothers (Profile 1) had higher incomes than other profiles. For COVID-related factors (model 2 in Table 2), compared to mothers in the High Acculturation group (Profile 2), mothers in the Low Acculturation group (Profile 1) reported that their child’s health provider recommended the COVID vaccine, had lower COVID vaccine mistrust, and intended to vaccinate their child concurrently and annually against COVID-19. Additionally, compared to mothers in the High Acculturation group (Profile 2), mothers in the Moderate Acculturation group (Profile 3) reported that their child's health provider recommended the COVID vaccine. Low Acculturation mothers (Profile 1) were more accepting of the current COVID-19 vaccine for their child than mothers in the Moderate Acculturation group (Profile 3), who in turn were more accepting of the COVID-19 vaccine for their child than High Acculturation mothers (Profile 2).

**Table 2:** Multinomial logistic regression model

		Profile 1 vs. Profile 2			Profile 3 vs. Profile 2			Profile 1 vs. Profile 3		
		β (*p*-value)	OR (SE)	CI	β (*p*-value)	OR (SE)	CI	β (*p*-value)	OR (SE)	CI
*Model 1*	Age of child	.14(.28)	1.15(.15)	.89–1.49	**.39(.02)**	**1.48(.25)**	**1.06–2.06**	–.25(.18)	.78(.15)	.54–1.12
	Insurance	–.52(.30)	.60(.30)	.22–1.60	–1.07(.09)	.34(.22)	.10–1.18	.55(.40)	1.74(1.14)	.48–6.32
	Income	**.63(.00)**	**1.88(.30)**	**1.38–2.56**	.03(.90)	1.03(.24)	.65–1.64	**.61(.02)**	**1.83(.46)**	**1.12–3.00**
*Model 2*	Provider’s recomm. COVID shot	**1.24(.00)**	**3.47(1.18)**	**1.78–6.75**	**1.65(.00)**	**5.21(2.26)**	**2.23–12.20**	–.41(.39)	.67(.32)	.26–1.69
	Covid Mistrust	**.48(.01)**	**1.62(.28)**	**1.15–2.28**	.36(.10)	1.43(.31)	.94–2.19	.12(.57)	1.13(.25)	.74–1.74
	Current COVID-19 intent	**2.45(.00)**	**11.61(4.52)**	**5.41–24.90**	.90(.11)	2.45(1.36)	.83–.7.29	**1.56(.01)**	**4.72(2.65)**	**1.58–14.19**
	Annual COVID-19	**.78(.02)**	**2.18(.74)**	**1.13–4.24**	.67(.11)	1.95(.81)	.86–4.42	.11(.80)	1.12(.51)	.46–2.71
Model 3	Provider’s recomm. Flu shot	**.97(.03)**	**2.63(1.16)**	**1.11–6.24**	.12(.81)	1.13(.55)	.43–2.94	.85(.16)	2.34(1.40)	.72–7.57
	Flu Mistrust	**.37(.02)**	**1.44(.33)**	**1.06–1.97**	.31(.20)	1.36(.23)	.85–2.19	.06(.83)	1.06(.28)	.63–1.79
	Current Flu intent	**1.02(.00)**	**2.78(.96)**	**1.41–5.45**	.88(.07)	2.41(1.18)	.92–6.30	.14(.80)	1.15(.65)	.38–3.49
	Annual Flu intent	**2.57(.00)**	**13.12(7.62)**	**4.21–40.95**	1.05(.08)	2.85(1.69)	.89–9.10	**1.53(.05)**	**4.61(3.54)**	**1.02–20.77**

*Note.* OR (SE) = Odds ratio and standard error. 95% confidence intervals (CI) were utilized to determine statistical significance. Bolded values were determined to be significant based on a confidence interval that does not cross 1.00

For influenza-related factors (model 3 in Table 2), similar to the COVID-19 model, compared to High Acculturation mothers (Profile 2), Low Acculturation mothers (Profile 1) reported that their child’s provider had recommended the flu vaccine, endorsed less flu vaccine mistrust, and intended to vaccinate their child concurrently and annually against the flu. Mothers in the Low Acculturation group (Profile 1) were more accepting of the seasonal flu vaccine than High Acculturation mothers (Profile 2) and Moderate Acculturation mothers (Profile 3).

We additionally conducted crosstab analyses to examine percentages among profiles with respect to COVID/influenza vaccination intent and provider’s recommendation for COVID and influenza vaccination (see Table 3). Relative to other profiles, mothers in the Low Acculturation group (Profile 1) reported higher income, had lower COVID vaccine mistrust, were more likely to have plans to vaccinate their child against COVID-19 and flu, and were more accepting of annual COVID-19 and flu vaccinations. High Acculturation mothers (Profile 2) had the greatest resistance to current COVID-19 vaccination and seasonal COVID and Influenza vaccinations. However, although Low Acculturation mothers (Profile 1) percentages were higher than those in the other profiles, only 68.4% of Low Acculturation mothers intended to have their child vaccinated against COVID-19. Provider’s recommendation of the COVID-19 vaccine was important. Twice as many Moderate Acculturation mothers (Profile 3) and Low Acculturation mothers (Profile 1) reported their doctor recommended vaccinating their young child against COVID-19 compared to High Acculturation mothers (Profile 2). Moreover, the majority of mothers in Low Acculturation group (Profile 1) and Moderate Acculturation group (Profile 3) would vaccinate their child annually against influenza compared to approximately half the mothers in High Acculturation group (Profile 2). Across profiles, the majority reported their providers had recommended vaccination against influenza for their child. Overall, High Acculturation mothers (Profile 2) were the most hesitant group for both COVID-19 and influenza.

**Table 3:** Unstandardized means, standard deviations, number and percentages of full sample and across profiles

	Range	Full Sample (*n* = 309)	Profile 1 (*n* = 79)	Profile 2 (*n* = 202)	Profile 3 (*n* = 28)
Profile Indicators					
Years in the US	11–14	13.32(1.03)	11.68(.05)	14.00(.00)	13.00(0.00)
Behavioral Acculturation	1–5	3.70(.87)	3.21(.83)	3.97(.78)	3.18(.68)
Psychological Acculturation	1–3	1.64(.49)	1.55(.38)	1.68(.54)	1.66(.38)
Cultural Medical Mistrust	1–5.8	3.11(.79)	3.27(.49)	3.02(.89)	3.39(.57)
Accessibility	1–6	4.21(1.05)	3.56(.98)	4.48(.97)	4.12(1.00)
Financial Security	1–3	2.05(.69)	2.41(.59)	1.89(.66)	2.14(.80)
Profile Correlates					
Age	1–4	2.35(1.04)	2.48(.93)	2.26(1.09)	2.68(.86)
Insurance^a^	1–2	287(92.9%)	72(91.1%)	191(94.6%)	24(85.7%)
Income^b^	1–3	2.11(.80)	2.41(.59)	2.01(.86)	2.07(.72)
Provider’s recommendation of COVID shot^c^	1–2	95(30.7)%	42(53.2%)	37(18.3%)	16(57.1%)
COVID vaccine Mistrust	1–5.9	3.32(1.02)	3.18(1.07)	3.37(1.00)	3.39(1.02)
Current COVID vaccination intent^c^	1–2	100(32.4%)	54(68.4%)	36(17.8%)	10(35.7%)
Seasonal COVID vaccination intent^c^	1–2	96(31.1%)	40(50.6%)	46(22.8%)	10(35.7%)
Provider’s recommendation of flu shot^c^	1–2	243(78.6%)	72(91.1%)	149(73.8%)	22(78.6%)
Flu Vaccine Mistrust	1–6	3.17(1.06)	3.05(1.04)	3.22(1.06)	3.21(1.16)
Current Flu vaccination intent^c^	1–2	239(77.3%)	78(98.7%)	136(67.3%)	25(89.3%)
Seasonal Flu vaccination intent^c^	1–2	208(67.3%)	74(93.7%)	113(54.3%)	21(75.0%)

*Note.* ^a^ indicates the percentage of Hispanic individuals that have insurance. ^**b**^ indicates the mean of income, which was recoded into three groups: 1 = less than $20,000, 2 = $20,000 to 29,999, 3 = $30,000 to 54,999. ^**c**^ indicates the percentage of provider’s recommendation of the COVID/flu and acceptance for current and seasonal COVID/flu shot

## Discussion

The continuing low COVID-19 vaccination rates among young Hispanic children and the declining rates of influenza vaccination stemming from the COVID-19 pandemic [59], highlight the need to better understand socio-cultural determinants influencing parental vaccine hesitancy among lower-income economically marginalized Hispanic parents. Guided by the cultural health belief model [14], this study applied a person-centered approach and identified three distinct profiles based on cultural and structural factors among economically marginalized Hispanic mothers associated with vaccine acceptance. The identification of these profiles emphasizes the point that lower-income Hispanic families are not a homogenous group and that tailored strategies are needed for families with different profiles to promote pediatric COVID-19 and flu vaccination.

Three unique profiles emerged among Hispanic mothers in our study differing in patterns of acculturation and degree of cultural medical mistrust, vaccination access, and financial security. Mothers in the Low Acculturation profile reported moderate cultural medical mistrust, low accessibility, and high financial security. By contrast, mothers in the High Acculturation profile reported low cultural medical mistrust, high accessibility, and low financial security. Mothers in the Moderate Acculturation group were midway between the other profiles, reporting high medical mistrust, low accessibility, and moderate financial security. Among profiles, the High Acculturation mothers were least accepting of COVID-19 and influenza vaccinations for their young children. The data point to acculturation as a potential risk factor for COVID-19 and influenza vaccine hesitancy. These findings are consistent with prior research suggesting that in some settings acculturation is associated with lower levels of vaccination and medical treatment readiness among Hispanic adults [60–62].

Consistent with prior research [63, 64], mothers in the Low and the Moderate Acculturation groups reported greater cultural medical mistrust than High Acculturation mothers. Yet, contrary to expectations, these mothers were also more vaccine accepting. Taken together, these findings suggest that healthcare decisions by less acculturated mothers, who may retain strong Hispanic values, such as *familism, respecto* and *confianza* (trust) [17, 65–68] may be driven by a responsibility to provide one’s children with medical care and an obligation to respect health care providers that override other obstacles such as concerns that Hispanics may not be treated as well as other groups by health care providers.

Mothers in the Low Acculturation and Moderate Acculturation profiles also indicated greater financial security but less access to vaccines for their children than High Acculturation mothers. This draws attention to a paradoxical finding across profiles: Financial security and accessibility were inversely related. While the reason behind this relationship is unclear, our data suggest that a heavier financial burden may not necessarily translate into a perception of greater accessibility challenges for vaccination. The majority of mothers in our study reported household annual incomes between $20,000—$40,000. However, most mothers (92%) reported their child was covered by health insurance. Thus, irrespective of income, insurance did not appear to be a barrier to pediatric vaccination. It is possible that economically marginalized parents who report greater financial security are working more hours and more jobs to alleviate financial burden, which in turn limits their time and ability to overcome barriers to taking their young child to the doctor’s office. These findings underscore the nuanced relationship between economic challenges and vaccine accessibility in this population.

The data also suggest that recommendations from a healthcare provider were an important factor in influencing vaccine acceptance in this sample. Twice as many mothers in the Low Acculturation and Moderate Acculturation profile, compared to the High Acculturation profile, reported that their provider had recommended the COVID-19 vaccine for their child. Interestingly, across profiles, mothers were more likely to accept influenza vaccination compared to acceptance of COVID-19 vaccination for their children. Consistent with the importance of COVID-19 provider recommendations, mothers also reported that providers were more likely to recommend the influenza vaccination.

Overall, the person-centered approach facilitated the exploration of how the interplay between cultural and structural variables influences vaccination intentions of Hispanic mothers regarding COVID-19 and influenza for their children. This methodological approach provided a unique perspective that complemented prior work utilizing variable-centered approaches to examine COVID-19 vaccine hesitancy among Hispanic mothers of children under five in the US [45]. However, this variable centered research while important in demonstrating factors influencing vaccine hesitancy, runs the risk of masking individual differences within populations. Through a person-centered approach, our findings indicated distinct individual differences in the combination of factors predicting vaccine hesitancy related to variations in mothers’ acculturation, medical mistrust, access to vaccines, and financial security. Thus, while both variable and person-centered approaches identify greater acculturation as a risk factor for vaccination hesitancy among economically marginalized Hispanic mothers, findings from the person-centered approach underscore how varying cultural and structural factors combine to influence differences in health behaviors.

Although this study has many strengths, a few limitations need to be considered. Our analysis met sample size criteria, but a greater sample size in future research is needed to further validate the profiles [69]. Second, the cross-sectional design of our study limited the ability to examine causal inferences related to profile correlates. Third, though we purposely collected data from women caregivers based on work suggesting that women are more responsible for health care decision-making for their children than men [70], the influence of men caregivers on family healthcare decisions is an important area of study to better understand the multi-dimensional aspect of pediatric COVID-19 and flu vaccination family decision-making. We selected Texas, Arizona and New Mexico because of their larger Hispanic populations and relatively low level of pediatric vaccine uptake compared to other states. However, since our mothers were predominantly Mexican, our findings may not be generalized to other Hispanic/Latina economically marginalized mothers in other regions of the US. In addition, across the general population during the period of data collection these states had more politically conservative attitudes toward vaccination than some other states, which may have influenced the hesitant attitudes of mothers who were more acculturated to Anglo values in their state [37]. Finally, data was collected during a period in which COVID-19 vaccination for children under 5 was first approved. Future research is needed to examine whether factors such as COVID-19 and influenza vaccine mistrust and healthcare providers’ recommendations have changed over time and whether the relationship between these factors and individual profiles remains consistent.

## Conclusion

This study is the first to apply the cultural health belief model and a person-centered approach to understand profile differences in pediatric COVID-19 and influenza vaccine acceptance among economically marginalized Hispanic families. The emergence of distinct profiles among lower income and economically marginalized Hispanic mothers, underscores the importance of exercising caution against public health approaches to vaccination uptake based on homogenous assumptions for this population and suggests shifting to a focus on person-centered strategies for improving the health of Hispanic children. Differences in the pattern of relationships between acculturation, cultural medical mistrust, accessibility, and financial security distinguished parents who were high and low in acceptability of vaccination their children against COVID-19 and influenza.

These findings suggest that public health strategies to increase pediatric vaccination will benefit from person-centered approaches tailored to the needs of different profile groups. For example, although mothers in the Low Acculturation group were more accepting of vaccinations and reported greater financial security, between 30 and 50% would not consider COVID-19 vaccination either currently or annually. They nonetheless also indicated less accessibility to vaccines. This suggests that public health strategies for these families should focus on how to increase access to vaccination sites and times at which such sites are open to accommodate for and reduce the burden of vaccination access for working parents and those with care-taking responsibilities in economically marginalized settings. The finding that acculturation is a risk factor for pediatric COVID-19 and influenza vaccination acceptance may also point to profile targeted interventions. Many mothers in the higher acculturation group were most vaccine hesitant and endorsed feelings of belongness to both Hispanic and Anglo cultures. This suggests that for this group of mothers, an effective way of enhancing vaccine acceptance may be community-based intervention programs emphasizing family roots in Hispanic values such as *familism, respect*, and *confianza.*

Finally, many parents in our study were unsure if their child had received a flu shot during infancy and recommendations by a provider were an important factor differentiating mothers who were or were not vaccine accepting. This suggests a critical need for a centralized public health system of record keeping for childhood vaccinations that reduces the burden on economically marginalized Hispanic parents who may not be aware of nor remember the multiple routine vaccinations their child received during early infancy and who may see different providers for pediatric visits. This underscores the importance of a public health infrastructure, intervention designs and medical training that include ways to enhance communication between healthcare providers and economically marginalized Hispanic mothers.

## Data Availability

Data supporting the findings of this study are available from the corresponding author C.B.F on request.
